# Global functionality and associated factors in the older adults followed by Home Care in Primary Health Care*

**DOI:** 10.1590/1518-8345.5026.3476

**Published:** 2021-10-29

**Authors:** Mariane Lurdes Predebon, Gilmara Ramos, Fernanda Laís Fengler Dal Pizzol, Naiana Oliveira dos Santos, Lisiane Manganelli Girardi Paskulin, Idiane Rosset

**Affiliations:** 1 Unimed Porto Alegre, Provimento de Saúde, Porto Alegre, RS, Brazil.; 2 Hospital Moinhos de Ventos, Unidade de Internação, Porto Alegre, RS, Brazil.; 3 University of Alberta, Faculty of Nursing, Edmonton, Alberta, Canada.; 4 Universidade Franciscana, Departamento de Enfermagem, Santa Maria, RS, Brazil.; 5 Universidade Federal do Rio Grande do Sul, Porto Alegre, RS, Brazil.; 6 Hospital de Clínicas de Porto Alegre, Coordenação do Grupo de Enfermagem, Porto Alegre, RS, Brazil.; 7 Hospital de Clínicas de Porto Alegre, Serviço de Enfermagem em Atenção Primária em Saúde, Porto Alegre, RS, Brazil.

**Keywords:** Geriatric Nursing, Aging, Activities of Daily Living, International Classification of Functioning, Disability and Health, Home Health Nursing, Primary Health Care, Enfermería Geriátrica, Envejecimiento, Actividades de la Vida Diaria, Clasificación Internacional del Funcionamiento, de la Discapacidad y de la Salud, Cuidados de Enfermería en el Hogar, Atención Primaria de Salud, Enfermagem Geriátrica, Envelhecimento, Atividades Cotidianas, Classificação Internacional de Funcionalidade, Incapacidade e Saúde, Enfermagem Domiciliar, Atenção Primária à Saúde

## Abstract

**Objective:**

to analyze the association of global functionality with the main functional systems and the sociodemographic variables of older adults followed by Home Care in Primary Health Care.

**Method:**

a cross-sectional study with 124 older people developed through home interviews. Functionality was assessed by Basic Activities of Daily Living (Barthel) and Instrumental Activities of Daily Living (Lawton and Brody); the main functional systems were assessed using the Mini-Mental State Exam, by the Timed Up and Go test, by the Geriatric Depression Scale (15 items), and questionnaire with sociodemographic variables. Bivariate and multivariate analyses were applied (Poisson Regression).

**Results:**

46% of the older adults showed moderate/severe/total dependence for basic activities and instrumental activities had a median of 12. In the multivariate analysis, there was an association between moderate/severe/total dependence on basic activities with cognitive decline (p=0.021) and bedridden/wheelchair users (p=0.014). Regarding the dependence on instrumental activities, there was an association with age ≥80 years (p=0.006), single/divorced marital status (p=0.013), cognitive decline (p=0.001), bedridden/wheelchair (p=0.020), and Timed Up and Go ≥20 seconds (p=0.048).

**Conclusion:**

the decline in cognitive and mobility was associated with poor functionality in basic and instrumental activities. The findings highlight the need to monitor Home Care for these individuals and serve as guidelines for health actions.

## Abstract (es)

**Objetivo:**

analizar la asociación de la funcionalidad global con los principales sistemas funcionales y con variables sociodemográficas de los adultos mayores vinculados a la Atención Domiciliar de la Atención Básica.

**Método:**

estudio transversal con 124 adultos mayores realizado por medio de entrevistas domiciliares. La funcionalidad fue evaluada por las Actividades Básicas de Vida Diaria (Barthel) y por las Actividades Instrumentales de Vida Diaria (Lawton y Brody); los principales sistemas funcionales fueron evaluados con el Mini Examen del Estado Mental, el test *Timed Up and Go* y la Escala de Depresión Geriátrica (15 ítems); además, se utilizó el cuestionario con variables sociodemográficas. Se aplicaron análisis bivariados y multivariados (Regresión de Poisson).

**Resultados:**

46% de los adultos mayores poseían dependencia moderada/grave/total para las actividades básicas y presentaron mediana de 12 en las actividades instrumentales. En los análisis multivariados, hubo asociación entre dependencia moderada/grave/total en las actividades básicas con disminución cognitiva (p=0,021) y en postrado en cama o en silla de ruedas (p=0,014). En la dependencia de las actividades instrumentales hubo asociación con edad ≥80 años (p=0,006), estado conyugal soltero/divorciado (p=0,013) con disminución cognitiva (p=0,001), postrado en cama o en silla de ruedas (p=0,020) y *Timed Up and Go* ≥20 segundos (p=0,048).

**Conclusión:** la disminución del estado cognitivo y de la movilidad estuvo asociada con peor funcionalidad en las actividades básicas e instrumentales. Los hallazgos destacan la necesidad del acompañamiento de la Atención Domiciliar para esos individuos y sirven como orientadores de acciones de salud.

## Abstract (pt)

**Objetivo:**

analisar a associação da funcionalidade global aos principais sistemas funcionais e às variáveis sociodemográficas de idosos vinculados à Atenção Domiciliar da Atenção Básica.

**Método:**

estudo transversal com 124 idosos realizado por meio de entrevistas domiciliares. A funcionalidade foi avaliada pelas Atividades Básicas de Vida Diária (Barthel) e pelas Atividades Instrumentais de Vida Diária (Lawton e Brody); os principais sistemas funcionais foram avaliados pelo Mini Exame do Estado Mental, pelo teste *Timed Up and Go*, pela Escala de Depressão Geriátrica (15 itens), além de questionário com variáveis sociodemográficas. Aplicaram-se análises bivariada e multivariada (Regressão de Poisson).

**Resultados:**

46% dos idosos possuíam dependência moderada/elevada/total para as atividades básicas e apresentaram mediana de 12 nas atividades instrumentais. Na análise multivariada, houve associação entre dependência moderada/elevada/total nas atividades básicas com declínio cognitivo (p=0,021) e em acamado/cadeirante (p=0,014). Na dependência das atividades instrumentais houve associação com idade ≥80 anos (p=0,006), estado conjugal solteiro/divorciado (p=0,013), declínio cognitivo (p=0,001), acamado/cadeirante (p=0,020) e *Timed Up and Go* ≥20 segundos (p=0,048).

**Conclusão:**

o declínio da cognição e da mobilidade esteve associado à pior funcionalidade nas atividades básicas e instrumentais. Os achados ressaltam a necessidade do acompanhamento da Atenção Domiciliar para esses indivíduos e servem como norteadores de ações de saúde.

## Introduction

Home Care (HC) has strategic potential given the scenario of fast population aging and the increase in chronic diseases that impact the functionality of individuals. In the Brazilian Health System (Portuguese: *Sistema Único de Saúde*, SUS), HC is organized into three modalities: Home Care type 1 (HC1), which is intended for users who require care with less frequency and complexity, being the responsibility of Primary Health Care (PHC); Home Care type 2 (HC2) and Home Care type 3 (HC3), which comprise the *Melhor em Casa* (Best at Home) program, being the responsibility of specialized services called Home Care Service (HCS) (Portuguese: *Serviço de Atenção Domiciliar*, SAD)^(1)^.

HC1 is still insufficiently designed as to the systematization of the care provided, besides being scarcely addressed in published literature, when compared to HC2 and HC3. However, HC1 plays an important role in preventing worsening and health complications of individuals who find it hard to get to the health care service^(1)^. Although it is not directed only to the older adult, it is observed, in practice, that most users are over 60 years old, mostly in places with an older population.

The older adults inserted in HC1 have some health disability and, often, limitations in their functional abilities. In this sense, the assessment of global functionality and associated factors is essential for identifying changes, implementing interventions, and establishing personalized therapeutic goals^(2)^.

Global functionality is a comprehensive concept that encompasses the individual’s ability to manage his/her life and take self-care. It is the foundation of the health concept for the older adult and is anchored to the main functional systems of cognition, communication, mood, and mobility^(2)^. Studies conducted in Brazil, the United States, and England, with the older population in general, identified as factors associated with the decline in functional capacity^(3-5)^: illiteracy, older age (over 70 years old), having chronic diseases, cognitive decline, and not having social support, thus showing a complex network of multidimensional factors related to functionality.

Gerontological nursing in the community can take action on several fronts, such as the provision of psychological, nutritional, and physical support for the older adult, being able to dedicate especially to actions of global functionality and activities of daily living^(6-8)^. In this sense, an intervention study conducted in New Zealand by nurses specialized in gerontology assessed the global functionality of the older population in the community using the Barthel index and the instrumental activities scale by Lawton and Brody. The authors identified a statistically significant increase for the intervention compared to the control group (p=0.03) concerning physical therapy as a form of rehabilitation^(9)^. In this perspective, the global functionality must be related to the associated factors of older people monitored by HC1, as this knowledge may enable the planning and implementation of specific gerontological nursing interventions that contribute to prevent injuries, postpone, or rehabilitate detected disabilities, besides subsidizing care management. Further, the assessment of global functionality may be used to obtain health indicators and outline care plans for the older adults followed by HC1.

HC1 is near to the individuals monitored because it is the PHC’s responsibility since it keeps the bond, the integrality, and the continuity of care to its users, which allows for early intervention and monitoring of health conditions^(10)^. However, there is still much to be explored, especially regarding gerontological nursing and its systematization. It is necessary to expand knowledge and technologies in the pace of demographic and epidemiological transition, which requires services and health care teams prepared to serve the fast-growing older population, since there may be a significant percentage of older people dependent on care and consequently, HC, especially provided by SUS.

Given the above, the study aimed to analyze the association of global functionality with the main functional systems and the socio-demographic variables of older people followed by HC in PHC. This study contributes to the discussion on new health care practices, especially those aimed at gerontological nursing and on health policies aimed at the care of the older adult linked to HC1.

## Method

### Study Design, Scenario, Period, and Population

A cross-sectional analytical study, conducted in the Central Sanitary District (CSD) of the municipality of Porto Alegre (RS), Brazil, from October 2018 to April 2019. Among the Brazilian capitals, Porto Alegre is the capital with the highest proportion of older people and the CSD, besides being the most populous CSD in the municipality, also has the highest proportion of older people (n=60,371), representing approximately 28.5% of the older population in the municipality^(11)^. The CSD is composed of three PHC services that follow-up 227 users in HC1. Of this total, 225 are older adults, as shown in the records provided by health professionals in the respective services.

### Sample definition and selection criteria

To calculate the sample size, we used the WinPepi program, version 11.65. Based on a study conducted in the same city^(12)^, considering a significance level of 5%, the statistical power of 80%, a proportion of greater functional dependence estimated at 40%, and a minimum prevalence ratio of 1.7 to estimate associated factors the instruments that assess functional capacity, the total of 112 older people was reached. However, it was chosen to include the entire older population linked to the HC1 of the three services and, applying the inclusion and exclusion criteria, it totaled 124 older people.

The inclusion criteria were people aged ≥60 years old, followed by the HC1 of the CSD in the city of Porto Alegre. The exclusion criteria were older adults who were not reached after three attempts at telephone contact, on different times and days, and who were not at home in the attempt of a home visit; those unable to communicate verbally or through writing; or with a previous medical diagnosis of advanced dementia and institutionalized older adult. Such conditions were informed by the health care team based on the user’s medical record, or by the caregiver at the telephone or home visit.

### Data collection

Data collection was conducted through structured interviews, at the participants’ homes, conducted by nurses and undergraduate nursing students, previously trained. At first, the identification of users followed by the HC1 of the CSD in the city of Porto Alegre was developed through the records that had the contact information provided by the health care team. For those individuals who met the inclusion criteria, telephone contact was made to schedule an interview at home and, if telephone contact was not possible, there would be an attempt to go to the home. In this first approach, the eligibility criteria were confirmed and, therefore, 101 older people were excluded.

### Study Variables and Instruments Used to Collect Information

A questionnaire with the following sociodemographic data variables: age, biological sex, education, marital status, family income, living alone or with a companion, morbidities, and time of follow-up in HC1 was used. Also, the presence or absence of a caregiver was investigated, considering caregiver the person responsible for assisting or providing physical, emotional, and/or financial support, and may be an informal caregiver (family or unpaid friend) or formal caregiver (hired and paid to provide care)^(13)^. It should be noted that these data were self-reported.

Global functionality was measured by the functional ability to perform Basic and Instrumental Activities of Daily Living by the Barthel Index and the Lawton and Brody scale, respectively. The Barthel Index, a version validated for use in Brazil^(14)^, assesses the functionality in ten Basic Activities of Daily Living (BADL), with the score varying from 0 to 100. Scores below 20 indicate total dependence; from 20 to 35 indicates severe dependence; from 40 to 55, moderate dependence; from 60 to 99, slight dependence, and 100 is considered independent^(14)^. Also, we used the version of the Lawton and Brody Scale recognized by the Brazilian Ministry of Health^(15)^, widely applied for functional assessment of the older adult in PHC and clinical practice. This scale assesses nine Instrumental Activities of Daily Living (IADL), being assigned from one to three points to each activity (“Independent” - three points; “Needs assistance” - two points; “Unable” - one point). The higher the total score, the greater the older adult’s independence^(15)^. The author of the original scale and the version in the Brazilian Ministry of Health material^(15)^ do not propose a cutoff point. Thus, due to the asymmetric distribution found in the current study, for statistical analysis, it was chosen to classify the score in two categories, according to the median found (=12). They are: (a) older adults with a higher level of dependence for instrumental activities (scores below 12) and (b) older adults with a lower level of dependence for instrumental activities (scores equal to or greater than 12).

The main functional systems evaluated were cognition; communication; mobility; and mood. Cognition was assessed by the Mini-Mental State Examination (MMSE), a version adapted for use in Brazil^(16)^. The total score ranges from 0 to 30 points, and the cut-off point for cognitive decline is established according to education: 13 points for illiterates; 18 for elementary and middle school (1 to 8 incomplete years); and 26 for secondary education (8 or more years)^(16)^. To assess communication, we used questions 6 to 11 of the MMSE^(16)^, which include sentence repetition items and complying with orders that depend on the older adult’s hearing ability, besides writing and drawing. The score of these questions ranges from 0 to 9 points, the higher the score, the better the communication. The questions that assessed communication were not included in the multivariate analysis because they also belonged to the cognition scale with a strong correlation between these variables.

The reduced Geriatric Depression Scale (15 items), validated for use in Brazil, was used to assess mood^(17)^. The total score ranges from 0 to 15 points. Scores between 0 and 5 represent normal (absence of depressive symptoms), between 6 and 10 represent mild depressive symptoms, and between 11 and 15 represent severe depressive symptoms^(15,17)^.

The Brazilian version of the Timed Up and Go (TUG) test was used to assess mobility^(18)^. This instrument was applied according to the recommendations of the authors of the original instrument. Thus, the older person was told to sit in an armchair and to get up after the order “go”; walk forward to an established mark on the floor, at his/her usual speed; then, turn back and walk until he/she could sit in the same chair^(19)^. The cutoff points for this instrument were based on the original study (<10 seconds; 10 to 19 seconds; 20 to 29 seconds; ≥30 seconds)^(19)^. However, considering that this study sample were individuals who, in general, have mobility limitations (inclusion criteria in HC1), it was decided to classify the TUG in two categories: good mobility (time less than 20 seconds) and alteration in mobility (time equal to or greater than 20 seconds). It is noteworthy that the instrument was not used as an exclusion criterion, it was only used to assess the mobility of individuals who were able to perform the test. Therefore, the TUG was not applied to bedridden and wheelchair users, who were thus classified in the mobility category.

### Data Treatment and Analysis

The data collected were inputted twice in an Excel^®^ worksheet and later transferred to SPSS 21.0. To assess the factors associated with global functionality, bivariate and multivariate Poisson Regression analyzes were applied, analyzed dichotomously, with the highest risk outcome being: “moderate/severe/total dependence” in BADL and score <12 for IADL. The criterion for including the variable in the multivariate model was that it should have a p<0.10 value in the bivariate analysis. The measure of effect used was the Prevalence Ratio (PR), together with the 95% confidence interval. The 5% level of significance was adopted (p<0.05).

### Ethical aspects

The study was approved by the Research Ethics Committees of the *Hospital de Clínicas de Porto Alegre* (no. 2,740,678) and the Municipal Health Department of Porto Alegre (no. 2,900,696).

## Results

Most were females (75.8%), with an average age of 82.8 years (± 9.2). Table 1 shows the sociodemographic characteristics and the follow-up time in the sample’s HC1. It is worth mentioning that nearly half of the sample, 48.4%, had been followed up by HC1 for more than three years.

**Table 1:** Sociodemographic characteristics and follow-up time among older adults in HC1. Porto Alegre, RS, Brazil, 2021

Variables	n=124 (%)
Biological sex*	
Female	94 (75.8)
Male	30 (24.2)
Age group*	
60 - 69	15 (12.1)
70 - 79	25 (20.2)
80 - 89	54 (43.5)
90 - 99	30 (24.2)
Marital status*	
Married	33 (26.6)
Single/Divorced	32 (25.8)
Widow/Widowed	59 (47.6)
Education*	
0 - 4 years	56 (45.2)
5 - 8 years	37 (29.8)
>8 years	31 (25.0)
Family income* ^†‡^	
Up to 2 m.w.	43 (34.7)
3-5 m.w.	58 (46.8)
>5 m.w.	23 (18.5)
Source of income* ^‡^	
Retirement	99 (79.8)
Social Security Benefit	36 (29.0)
Support from Family	27 (21.8)
Follow-up time in HC1*	
<1 year	20 (16.1)
≥1 to 3 years	44 (35.5)
≥3 to 5 years	31 (25.0)
≥5 years	29 (23.4)

* Categorical variables (%);

† M.w. = 2018 minimum wage R$ 954.00;

‡ The older adult could have more than one source of income

With regard to caregivers, 77 older adults (62.1%) had a caregiver, of whom 59 (76.6%) were informal (family members or friends). Further, 84% of the older adults lived with someone, living with up to eight people in the same household. The median of morbidities was 3 (2-4). Regarding global functionality, according to the data presented in Table 2, only 15 (12.1%) were independent for BADL and 52 (41.9%) had slight dependence on BADL. While in IADLs, the median found was 12, and of the sample of 124 older people, 73 (58.9%) had a lower level of dependence for IADLs. As for the main functional systems, 61 older adults (49.2%) had cognitive decline, had an average of 7.1 in communication, close to the highest score evaluated, 61 (49.2%) stated having depressive symptoms (mild or severe), of which 11.3% were severe, 49 (39.5%) were bedridden/wheelchair users and, of those who walked (n=75), more than half (64%) had TUG ≥20 seconds.

**Table 2:** Global functionality and the main functional systems - cognition, communication, mood, and mobility among older adults. Porto Alegre, RS, Brazil, 2021

Variables	n=124 (%)
BADL - Barthel*	
Independence	15 (12.1)
Slight dependence	52 (41.9)
Moderate dependence	15 (12.1)
Severe dependence	19 (15.3)
Total dependence	23 (18.6)
IADL - Lawton and Brody^†^	12 (10 - 21)^‡^
IADL - Lawton and Brody<12^‡^	51(41.1)
IADL - Lawton and Brody≥12^‡^	73(58.9)
COGNITION - cognitive decline*	
Without	63 (50.8)
With	61 (49.2)
COMMUNICATION^†^	7.1 ± 2.0
MOOD - depressive symptoms*	
Normal	63 (50.8)
Mild	47 (37.9)
Severe	14 (11.3)
MOBILITY*	
TUG<20 seconds (n=27)	27 (21.8)
TUG≥20 seconds (n=48)	48 (38.7)
Bedridden/wheelchair user	49 (39.5)

* Categorical variables (%);

† Continuous variables [mean and standard deviation or median and interquartile range (P25-P75)];

‡ Score less than 12: older people with a higher level of dependence for instrumental activities; a score equal to or greater than 12: older people with a lower level of dependence for instrumental activities

Table 3 presents the bivariate analysis of factors associated with moderate/severe/total dependence in BADLs and with a score lower than 12 in IADLs. It is worth noting that the variables with p<0.10 in the bivariate analysis (Table 3) went to the multivariate model. In this bivariate analysis perspective, the functional communication system stands out, which clarified that for each extra score in its assessment, the risk of moderate/severe/total dependence on BADLs decreased by 16% and 18% of greater dependence on IADLs.

**Table 3:** Bivariate analysis of the association of changes in global functionality with the main functional systems and sociodemographic variables among the older adults. Porto Alegre, RS, Brazil, 2021

Variables (n=124)	BADL			IADL		
	Dependence* n=57 (%)^§^	PR^†^ (CI 95%)	p	<12^‡^ n=64(%)^§^	PR^†^ (CI 95%)	p
Biological sex						
Male (n=30)	12 (40.0)	1.00		12 (40.0)	1.00	
Female (n=94)	45 (47.9)	1.20 (0.74-1.95)	0.469	52 (55.3)	1.38 (0.86 - 2.22)	0.180
Age group						
<80 years (n=40)	14 (35.0)	1.00		13 (32.5)	1.00	
≥80 years (n=84)	43 (51.2)	1.46 (0.91 - 2.34)	0.114	51 (60.7)	1.87 (1.16 - 3.02)	**0.010**
Education						
0 - 4 years (n=56)	27 (48.2)	0.83 (0.55 - 1.24)	0.367	33 (58.9)	1.02 (0.70 - 1.47)	0.938
5 - 8 years (n=37)	12 (32.4)	0.56 (0.32 - 0.97)	**0.039**	13 (35.1)	0.61 (0.36 - 1.03)	**0.063**
>8 years (n=31)	18 (58.1)	1.00		18 (58.1)	1.00	
Live alone						
Yes (n=20)	2 (10.0)	0.19 (0.05 - 0.71)	**0.014**	2 (10.0)	0.17 (0.05 - 0.63)	**0.008**
No (n=104)	55 (52.9)	1.00		62 (59.6)	1.00	
Marital status						
Married (n=33)	12 (36.4)	1.00		13 (39.4)	1.00	
Single/Divorced (n=32)	14 (43.8)	1.20 (0.66 - 2.19)	0.545	15 (46.9)	1.19 (0.68 - 2.09)	0.544
Widow/Widowed (n=59)	31 (52.5)	1.45 (0.87 - 2.41)	0.159	36 (61.0)	1.55 (0.97 - 2.48)	**0.068**
Family income^||^						
1 - 2 m.w. (n=43)	16 (37.2)	0.54 (0.33 - 0.86)	**0.010**	18 (41.9)	0.54 (0.35 - 0.81)	**0.003**
3 - 5 m.w. (n=58)	25 (43.1)	0.62 (0.42 - 0.93)	**0.019**	28 (48.3)	0.62 (0.44 - 0.87)	**0.006**
>5 m.w. (n=23)	16 (69.6)	1.00		18 (70.3)	1.00	
Cognitive decline						
Without (n=63)	16 (25.4)	1.00		15 (23.8)	1.00	
With (n=61)	41 (67.2)	2.65 (1.67 - 4.18)	**<0.001**	49 (80.3)	3.37 (2.13 - 5.34)	**<0.001**
Communication	-	0.84 (0.79 - 0.90)	**<0.001**	-	0.82 (0.78 - 0.87)	**<0.001**
Depressive symptoms						
Normal (n=63)	22 (34.9)	1.00		24 (38.1)	1.00	
Mild (n=47)	26 (55.3)	1.58 (1.04 - 2.42)	**0.033**	27 (57.4)	1.51 (1.01 - 2.25)	**0.044**
Severe (n=14)	9 (64.3)	1.84 (1.10 - 3.08)	**0.020**	13 (92.9)	2.44 (1.72 - 3.45)	**<0.001**
Mobility						
<20 seconds (n=27)	1 (3.7)	1.00		1 (3.7)	1.00	
≥20 seconds (n=48)	18 (37.5)	10.1 (1.43 - 71.7)	**0.020**	24 (50.0)	13.5 (1.93 - 94.3)	**0.009**
Bedridden/wheelchair user (n=49)	38 (77.6)	20.9 (3.04 - 144)	**0.002**	39 (79.6)	21.5(3.12 - 147)	**0.002**

* Moderate, severe, and total dependence in the Barthel Index;

† PR = Prevalence Ratio;

‡ A score of less than 12 on the Lawton and Brody scale (older adults with a higher level of dependence for instrumental activities);

§ Percentage referring to the total sample (n=124);

|| m.w. = 2018 minimum wage R$ 954.00

Table 4 shows the findings of the multivariate analysis of factors associated with changes in global functionality. The statistically significant association of moderate/severe/total dependence on BADL with cognitive decline (p=0.021) and total mobility restriction (bedridden and wheelchair users) (p=0.014) was established. While, in IADLs, the association of scores below 12 with age greater than or equal to 80 years (p=0.006), single/divorced marital status (p=0.013), cognitive decline (p<0.001) was kept, mobility over 20 seconds through the TUG (p=0.048), and bedridden/wheelchair user (p=0.020).

**Table 4:** Multivariate analysis of the association of changes in global functionality to the main functional systems and sociodemographic variables among the older adults. Porto Alegre, RS, Brazil, 2021

Variables	BADL*		IADL^†^	
	PR^‡^(CI 95%)	p	PR^‡^(CI 95%)	p
Age group				
<80 years (n=40)	-	-	1.00	
≥80 years (n=84)	-	-	1.58 (1.14 - 2.18)	**0.006**
Education				
0 - 4 years (n=56)	0.88 (0.59 - 1.29)	0.498	1.02 (0.74 - 1.41)	0.903
5 - 8 years (n=37)	0.72 (0.45 - 1.17)	0.190	0.74 (0.51 - 1.07)	0.112
>8 years (n=31)	1.00		1.00	
Live alone				
Yes (n=20)	0.40 (0.15 - 1.06)	0.066	0.38 (0.13 - 1.12)	0.078
No (n=104)	1.00		1.00	
Marital status				
Married (n=33)	-	-	1.00	
Single/Divorced (n=32)	-	-	1.58 (1.10 - 2.26)	**0.013**
Widow/Widowed (n=59)	-	-	1.31 (0.90 - 1.90)	0.157
Family income^§^				
1 - 2 m.w. (n=43)	0.78 (0.53 - 1.15)	0.209	0.73 (0.53 - 1.01)	0.060
3 - 5 m.w. (n=58)	0.76 (0.54 - 1.08)	0.125	0.88 (0.67 - 1.15)	0.336
> 5 m.w. (n=23)	1.00		1.00	
Cognitive decline				
Without (n=63)	1.00		1.00	
With (n=61)	1.66 (1.08 - 2.54)	**0.021**	2.23 (1.54 - 3.23)	**<0.001**
Depressive symptoms				
Normal (n=63)	1.00		1.00	
Mild (n=47)	1.41 (0.95 - 2.09)	0.089	1.26 (0.91 - 1.75)	0.160
Severe (n=14)	1.04 (0.70 - 1.56)	0.833	1.25 (0.96 - 1.63)	0.093
Mobility				
<20 seconds (n=27)	1.00		1.00	
≥20 seconds (n=48)	6.10 (0.87 - 42.7)	0.069	6.77 (1.01 - 45.3)	**0.048**
Bedridden/wheelchair user (n=49)	11.5 (1.66 - 80.5)	**0.014**	9.56 (1.43 - 64.2)	**0.020**

* Moderate, high, and total dependence in the Barthel Index;

† Score less than 12 on the Lawton and Brody scale;

‡ PR = Prevalence Ratio;

§ m.w. = 2018 minimum wage R$ 954.00

## Discussion

Associations between moderate/severe/total dependence on BADL with cognitive decline and mobility problems were observed in the older adults followed by HC1. While in IADLs, there was an association between greater dependence at age ≥80 years, single/divorced marital status, cognitive decline, mobility problems, and TUG≥20 seconds. It is observed that the decline in cognition and mobility was associated with worse functionality in BADL and IADL, showing the need to protect these systems to maintain independence in the performance of their activities.

Most of the sample was female, as in other studies conducted in Brazil, United States, and China with older adults under HC^(20-22)^. As for age, more than half of the sample was 80 years old or over, older than the age group found in another study developed in low-income communities in the same municipality with older adults under HC^(20)^ and similar to a cross-sectional study conducted in the United States with older people followed by HC^(23)^. There may be a chance that people in a less fortunate socioeconomic condition, associated with other factors such as chronic illnesses, clinical conditions, a propensity to fall, and limited ability to perform physical exercises, develop dependence for the performance of activities and need care at home earlier^(24-25)^.

About half of the sample (48.4%) had been followed up by HC1 for more than three years. The longer the follow-up time, the greater the bond between professional and user, which may encourage care to keep its functionality. Japanese and European studies highlight the growing need for HC with an emphasis on the quality of life of the older adult, suggesting that, to achieve this purpose, changes in functionality must happen, besides investing in actions aimed at social engagement, cognitive development, and mental health^(25-27)^. Still, a systematic review and meta-analysis study of prospective cohort studies highlight that the early assessment of frailty can effectively prevent frailty-induced disability in the older adult(6). It should be observed that of the participants in the present study, 67 older people (54%) were independent or had slight dependence on BADLs, and 73 (58.9%) had a low level of dependence on IADLs, indicating the importance of early intervention by health professionals in the older population of HC1.

Regarding the need to have a caregiver, to live with somebody, and about the number of morbidities, it was observed that more than half of the older adults had an informal caregiver, lived with somebody, and with a median of three morbidities. The need to have a caregiver with advancing age is an event that has been discussed in national and international studies^(13,28-29)^. More specifically, informal care is the main type of care for the older adult in many countries^(30)^, being understood as a fundamental pillar for guaranteeing and maintaining HC for the frail older adult^(28,31)^. Thus, according to a systematic review that aimed to identify the preferences and needs on the organization of informal care in 44 studies from 17 different countries, demographic, financial, and cultural aspects of each circumstance must be considered, as these they may influence the preferences and needs of informal caregivers^(28)^. Therefore, the support network must be strengthened, especially HC1, to assist both the informal caregiver and the older adult with functional limitations at home^(13,29)^.

As for global functionality, more than a third of the sample showed slight dependence on BADLs and greater dependence on IADLs (with a low median). This characterization is expected for follow-up HC1^(2)^, although the percentage of these data was not known for that specific population. In natural aging, the individual first have a decrease in the ability for performing complex activities, gradually increasing the need for family and social support in other tasks^(4)^.

Until recently, these older people received care only in the health service, to which they often had to travel bearing transport costs, while others only accessed such services through emergencies. In this sense, HC1 contributes to promote access to health. Therefore, the interventions provided must be directed towards the prevention of health problems, treatment, palliation, and rehabilitation, considering the assessment of the socioeconomic context, individually^(2)^.

Regarding the cognition functional system, the percentage of cognitive decline in studies that used MMSE was higher than that found in a cross-sectional study with older community members from seven Brazilian cities, in which 65.1% of the participants were younger than 75 years old, and only 24.8% had cognitive decline^(32)^. It was also greater than in an American cohort study with older people with an average baseline age of 90.3 ± 6.3 years, in which only 25% of these older people had cognitive decline^(33)^. These data highlight the specific limitations of older people who followed up by HC. Also, a South Korean study conducted with older people from the community informs that cognitive decline is a factor that may influence the frailty in older population^(34)^.

A South African study with focus groups conducted with older residents in a community, from three areas (high, medium, and low income) describes that communication is necessary for the older adults to understand their health condition and have adherence to the care plan presented^(35)^. Thus, as for the communication functional system, the average was close to the upper limit evaluated, indicating that the sample had good communication through language, writing, drawing, and listening. Another study with the older population that uses the internet in the United Kingdom and Australia shows that digital technology can make life easier for the older adult, once it allows overcoming physical barriers to access services, however, older people still have difficulties in dealing with new technologies^(36)^. As an example of the contribution of technology to the older adult, are the need for actions of inclusion and guidance for the use of communication applications such as WhatsApp, applications developed for reminders (such as remembering to drink water), and applications to stimulate them (such as memory games or yoga practice). Nevertheless, it is necessary to guide the older adult and their caregivers, who are sometimes also older adults, for the use of these technologies, especially robotics, which may qualify and improve the assistance provided. In this perspective, a Canadian study that proposed a remote assessment for older residents of the community during the COVID-19 pandemic indicates that, in cases where the older adult have difficulty using the technology, they can be assisted by someone else, such as a health professional or a caregiver^(37)^.

Regarding the mood functional system, about half of the sample had mild or severe depressive symptoms, which indicated the need for active listening approaches, with a psychosocial focus for these individuals. This percentage of older people with depressive symptoms was higher than that found in other national studies with older people in the community who applied the same assessment tool^(38-39)^. This finding may be related to the limitation to leave the house, which is a frequent concern of users of HC1. Depressive symptoms may result in indifference in performing activities of daily living^(40)^. Further, a cross-sectional Canadian study of individuals with work-related musculoskeletal disorders suggests that fatigue contributes to disability and that a significant decrease in fatigue can be achieved through psychosocial interventions that promote the individual’s rehabilitation, thereby lessening the severity of depressive symptoms^(41)^. In this sense, health professionals must be attentive to these circumstances, promoting activities aimed at reducing depressive symptoms, without neglecting the natural aging process.

Mobility was the functional system in which the sample had great limitations, as more than a third were bedridden/wheelchair users, and, of those who could walk, more than half had a time equal to or greater than 20 seconds in the TUG. Mobility problems, found in the present study, evidenced by the longer time for the performance of the TUG, work as a warning for preventive actions mainly because they increase the propensity for falls^(18)^. This change in mobility and its repercussions on social participation may be related to the depressive symptoms observed in almost half of the sample.

Regarding the factors associated with global functionality, sociodemographic characteristics did not show a statistically significant association in the multivariate analysis with moderate/severe/total dependence on BADL, diverging from other national studies with older people, in general, that used the Katz Index^(3-4)^. However, with a score of less than 12 in the IADL, a significant association was found in the age group equal to or older than 80 years (p=0.006) and the single/divorced marital status (p=0.013), showing that, with advancing age, the ability to perform more complex activities is lost. This finding is similar to a cross-sectional study with 313 older people from Portugal, which showed aged people without much concern with self-care activities, as they feel that it is not worth living, thus presenting a greater functional dependence^(42)^.

In the multivariate analysis, there was a significant association of a greater functional dependence in BADL and IADL with the cognition and mobility functional systems. This finding may be related to neurodegenerative diseases and their higher prevalence at older ages. Cognitive decline showed a similar association to other Brazilian studies with older people, in general, which used MMSE^(3,38)^. A cross-sectional study developed in four places in Canada and Latin America with 1,071 participants living in the community (aged 64-75 years) highlights the need for interventions focused on the cognitive and physical performance of the older adult, to prevent disabilities^(43)^. In this study, older adults with cognitive decline presented about 1.7 times the probability for those who do not have a cognitive decline to have moderate/severe/total dependence on BADLs, and 2.2 times to have a greater dependence on IADLs, showing the great interference of cognition in the performance of activities. The prevalence ratio was higher in more complex activities (IADL), because, to perform them, there is a requirement for greater cognitive ability, as shown in other studies^(4,43)^.

Regarding the change in mobility, being a bedridden/wheelchair user was associated with greater dependence on BADL and IADL. The association found emphasizes the need for a personalized care plan aimed at the implications of altered or mobility problems in the performance of activities, aiming at rehabilitating and/or preventing complications, such as pressure injuries and immobility syndrome^(2)^. Furthermore, time greater than or equal to 20 seconds in the TUG was associated with greater dependence on IADL (p=0.048), highlighting that the more complex activities, vital to have an independent routine in the community, may require better performance of the main functional systems. A Mexican cross-sectional study with 146 older people who used the Lawton and Brody Scale to assess IADL and the GAITRite system, a software connected to a portable walkway to assess mobility, showed that walking speed, cadence, and step length are variables that impact IADL^(44)^.

The communication in the bivariate analysis indicated that for each additional point in its assessment, the risk of moderate/severe/total dependence on BADLs decreased by 16% and 18% of greater dependence on IADLs. This finding underlines the importance of health professionals to develop actions aimed at stimulus communication and prevention, encompassing vision, hearing, and speech. A randomized clinical trial with 150 older people aged 65 to 98 years old verified that it is important to encourage the older adult to use technologies mainly because some are designed to facilitate the use by aged individuals, such as the PRISM computer system, developed to be more useful and easy, and may benefit the older adult’s memory, mood, in addition to strengthening their support network^(45)^.

In the multivariate analysis, the mood functional system did not show a significant association with greater dependence, diverging from other Brazilian studies with larger samples of older people in the community that employed the Lawton and Brody, Katz, and the reduced Geriatric Depression scales^(46-47)^. However, we emphasize the importance of professionals to intervene early, enabling family members to be alert to depression symptoms and to communicate to the health care team, to minimize dependence on the performance of functional capacity and impact on morbidities in the older adult^(46)^.

The findings of this study allow increasing the view of the HC1’s potential in the face of new health demands, especially in the monitoring of the older adult with functional limitations. International theoretical and research studies suggest that more efforts are needed to implement and coordinate HC services and, also, to guarantee good practices, with a view to the quality of care and health conditions at a fair cost for users with disabilities, to promote well-succeeded aging^(48-49)^. Thus, it is considered that, with the fast population aging and the consequent increase in demands on health care services for the older adult, especially for HC, it is necessary that nurses working in PHC improve knowledge directed to care in geriatrics and gerontology.

In clinical practice, it is observed that a full geriatric evaluation, with a suitable anamnesis and approach of the main functional systems, evaluated in this study, associated with the elaboration of a care plan focused on this population, considering that continuing this care allows the delay of health problems and the subsequent increase in the quality of life of the older adults^(50)^. Moreover, it reduces costs for the healthcare system and the stigma associated with disability^(50)^. It should be noted that HC1 is present in care management, identifies needs early, strengthens bonds and the formal and informal support network, thus expanding access to health.

This study presents as a limitation the cross-sectional design, not allowing causal inferences. In addition, the findings cannot be generalized, because of the specific characteristics of a given region and for not having included older people without communication skills and/or with advanced dementia.

New intervention studies are suggested to obtain more subsidies to assess the global functionality of older people monitored by HC1 as an outcome, to expand the discussion, to add well-being to longevity, and provide support to the older adults, family members, and/or caregivers.

## Conclusion

The decline in cognition and mobility functional systems was associated with worse functionality concerning basic and instrumental activities of daily living, while socio-demographic variables age ≥80 years and single/divorced marital status were associated with worse functionality to IADLs. The findings highlight the need to monitor HC1 for these individuals and work as important guidelines for planning and implementing health interventions aimed at keeping the functionality and well-being of the older population.

The management of care and gerontological evaluation of the older adult in the HC of PHC preserve the bond of the older adult with the service and improve the expansion of health maintenance and recovery actions, prevention of health problems and readmissions, as well as health promotion in the home environment. In this sense, based on the results from this study, the nurse will be able to evidence health indicators, besides developing personalized care plans for the older adult in PHC, in the home context.
